# TF Target Mapper: A BLAST search tool for the identification of Transcription Factor target genes

**DOI:** 10.1186/1471-2105-7-120

**Published:** 2006-03-08

**Authors:** Sebastiaan Horsman, Michael J Moorhouse, Victor CL de Jager, Peter van der Spek, Frank Grosveld, John Strouboulis, Eleni Z Katsantoni

**Affiliations:** 1 Department of Cell Biology, Erasmus Medical Center, Dr Molewaterplein 50, 3015GE Rotterdam, The Netherlands; 2 Department of Bioinformatics, Erasmus Medical Center, Dr Molewaterplein 50, 3015GE Rotterdam, The Netherlands; 3 Foundation for Biomedical Research of the Academy of Athens, Hematology Laboratory, Soranou tou Ephessiou 4, 115.27 Athens, Greece; 4 Present address : Department of Virology, Erasmus Medical Center, Dr Molewaterplein 50, 3015GE Rotterdam, The Netherlands; 5 Present address : NBIC, Toernooiveld 1, 6525 ED, Nijmegen, The Netherlands

## Abstract

**Background:**

In the current era of high throughput genomics a major challenge is the genome-wide identification of target genes for specific transcription factors. Chromatin immunoprecipitation (ChIP) allows the isolation of in vivo binding sites of transcription factors and provides a powerful tool for examining gene regulation. Crosslinked chromatin is immunoprecipitated with antibodies against specific transcription factors, thus enriching for sequences bound in vivo by these factors in the immunoprecipitated DNA. Cloning and sequencing the immunoprecipitated sequences allows identification of transcription factor target genes. Routinely, thousands of such sequenced clones are used in BLAST searches to map their exact location in the genome and the genes located in the vicinity. These genes represent potential targets of the transcription factor of interest. Such bioinformatics analysis is very laborious if performed manually and for this reason there is a need for developing bioinformatic tools to automate and facilitate it.

**Results:**

In order to facilitate this analysis we generated TF Target Mapper (Transcription Factor Target Mapper). TF Target Mapper is a BLAST search tool allowing rapid extraction of annotated information on genes around each hit. It combines sequence cleaning/filtering, pattern searching and BLAST searches with extraction of information on genes located around each BLAST hit and comparisons of the output list of genes or gene ontology IDs with user-implemented lists. We successfully applied and tested TF Target Mapper to analyse sequences bound in vivo by the transcription factor GATA-1. We show that TF Target Mapper efficiently extracted information on genes around ChIPed sequences, thus identifying known (e.g. *α-globin *and *ζ-globin*) and potentially novel GATA-1 gene targets.

**Conclusion:**

TF Target Mapper is a very efficient BLAST search tool that allows the rapid extraction of annotated information on the genes around each hit. It can contribute to the comprehensive bioinformatic transcriptome/regulome analysis, by providing insight into the mechanisms of action of specific transcription factors, thus helping to elucidate the pathways these factors regulate.

## Background

In the current era of high throughput genomics there is a need for bioinformatic tools that are able to: 1. Automate and facilitate the storage and handling of large numbers of sequences and 2. Mine and decipher information contained therein. The interpretation of such data can provide new insight into sequence-function relationships and transcriptional/post-transcriptional regulatory mechanisms. A major challenge today is the genome-wide identification of target genes/regulatory elements for specific transcription factors. Chromatin immunoprecipitation (ChIP) allows the isolation of in vivo binding sites of transcription factors and is a powerful tool for examining gene regulation [1]. In ChIP, crosslinked chromatin is immunoprecipitated with antibodies against specific transcription factors, thus enriching for sequences bound in vivo by these factors in the immunoprecipitated DNA. Cloning and sequencing the ChIPed DNA allows the identification of novel transcription factor target genes. Routinely, thousands of such sequenced clones are used in BLAST searches to map their exact location in the genome. Information on the genes around each hit then needs to be extracted to identify potential targets of the specific transcription factor of interest. Furthermore, specific arrangements of combinations of transcription factor binding sites are commonly found in the vicinity of genes involved in a specific function or pathway. Information on specific combinations of transcription factor binding sites on user submitted sequences also needs to be extracted, as it strengthens the prediction for a sequence being real or background.

## Implementation

The web front-end is programmed in PHP (v4.3) [2] running on an Apache WWW Server (v1.3) and forms an interactive layer between the user and the underlying analysis processes. All analysis data is stored in a MySQL database (v4.0) [3]. The background running processes are programmed in Perl (v5.8) [4]. Background running processes include sequence cleanup (vector cleanup and repeat removal using RepeatMaskerOpen 3.0 [5]), BLAST/Ensembl searches, creation of sequence images including transcription factors sites and hit visualization. For transcription factors binding sites identification, TRANSFAC Matrix tables [6] are used and converted to standard IUPAC codes using BioPerl [7]. The IUPAC text string is then used as a regular expression to match to the supplied sequence. For DNA manipulation, administering repeat removal using RepeatMasker [5], running BLAST searches and parsing the results the BioPerl libraries are used [7]. Nucleotide sequence comparison searches (BLAST queries) are performed with a local version of the NCBI BLAST program running the blastn algorithm [8]. Visualization of hit positions relative to the mouse genome are presented on a clickable chromosome ideogram, using cytogenetic banding data from Ensembl Table Browser [9]. For extraction of gene identifiers, descriptions and database cross-links from Ensembl and parsing the retrieved results, the Ensembl Perl API is used [10].

## Results

### General description of TF Target Mapper tool

**Figure 1:**
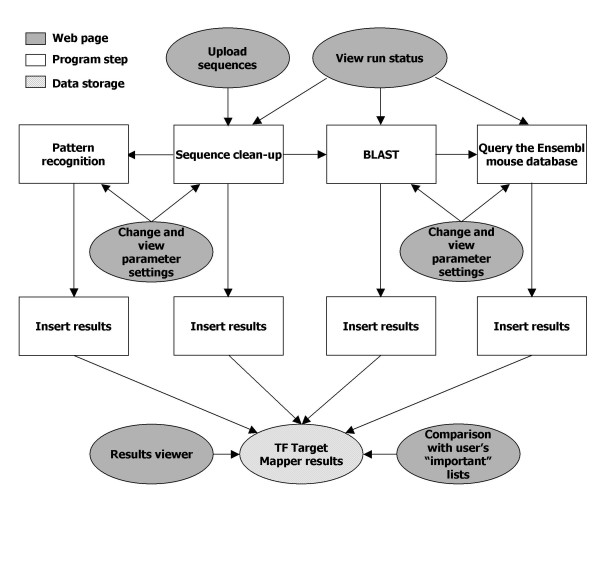
**TF Target Mapper application flowchart** :  Simplified flowchart of the TF Target Mapper application, summarizing its basic functions.

In order to facilitate the analysis of large sequence data collections of cloned DNA obtained from chromatin immunoprecipitations we generated a software tool called TF Target Mapper (Transcription Factor Target Mapper). This entails five functions (Figure 1, Additional File 1): 1. Cleaning/filtering of sequences: During this step large sequence data collections are uploaded and cleaned from vector sequences and repetitive elements. 2. Pattern recognition: Clean sequences are analyzed for specific transcription factor binding sites and their combinations. 3. BLAST searches: Clean sequences are used in BLAST searches to identify their exact location in the mouse genome. 4. Retrieval of information around each BLAST hit: Information within a user selectable window around each hit is extracted and linked to external databases. 5. Comparison of results with lists imported by the user: The output lists (genes and gene ontology IDs) are compared with user-supplied custom lists.

TF Target Mapper has support for multiple users, thus data can be compartmentalized into projects/individual investigations. After registering and logging in, the user can view the welcome page with information on the contents of the database (total number of sequences, BLAST hits, BLAST HSPs, Ensembl genes and the most recent genome and Ensembl versions in which the BLAST searches have been performed).

### Details

#### 1. Cleaning/filtering of sequences

Cleaning allows the user to strip the submitted sequences of vector sequence contamination and repetitive elements. Since cloned chromatin immunoprecipitated DNA fragments are usually small in size, vector sequences might be present on both sides of the inserts/submitted sequences and should be stripped before the BLAST searches. The user can upload specific vector sequences and set various parameters like vector clipping minimum match and score and insert length threshold. The stripping of the vector sequences is implemented by using the Cross_Match program [11]. Most cloning strategies for immunoprecipitated DNA involve digesting the DNA with restriction endonucleases prior to cloning into a vector with compatible restriction ends. This raises the possibility of unrelated fragments ligating to each other and cloned together. To counter this possibility, we inserted an option for the in silico digestion of uploaded sequences with the restriction enzyme(s) used in cloning the DNA, followed by separate BLAST analysis of the co-ligated sequences. The user can select whether or not to digest the sequence with a restriction enzyme of choice. Specific restriction enzymes of interest can be uploaded. The vector-free sequences are subsequently scanned for repetitive elements using Repeat Masker [5]. An option for omitting this step also exists. Sequences cleaned from vector and repeats are stored in a database. These filter features restrict the BLAST searches to repeat and vector free sequences, resulting in a drastic reduction in false positive hits.

#### 2. Pattern recognition

Pattern recognition allows the user to identify specific combinations of transcription factor binding sites in the cleaned input sequences. The user can upload transcription factors of interest as a file with TRANSFAC Matrix entries from the TRANSFAC database [6]. TF Target Mapper converts these entries to IUPAC codes and then expands them to a regular expression which is used to search the input sequence. The exact location of the sites in the input sequences can then be visualized in graphics generated using the BioPerl modules. Visualization of specific combinations of hematopoietic transcription factor binding sites strengthens the prediction of a sequence being real or background and might provide a first indication of potentially "interesting" sequences.

#### 3 & 4. BLAST searches-retrieval of information from Ensembl

BLAST searches allow the user to identify the exact location of the sequence in the genome [12,13]. Clean sequences are BLASTed against the mouse genome using the NCBI BLAST program and the outcome (hit/HSP positions, E-value, score percent identities, length, start/end query, chromosome) is stored in a database. The user can select and set various BLAST parameters (from the parameter settings page), such as e-value, gapped alignment, word size, matrix and maximum number of HSPs. Before the run starts the Run Info table is initialized allowing the user to check the status of the BLAST run. Retrieval of annotated information around each BLAST hit allows the user to extract information on the genes around the hit that may include potential targets of the transcription factor of interest. The Ensembl database [9,14] is queried with the hits of the BLAST run and results on the annotation of genes upstream and downstream of each hit are stored in a database (the length of the window around each hit is variable and can be set in the BLAST parameter settings page). The position of hits on a mouse chromosome ideogram can be also visualized.

#### 5. Comparisons

The output list of genes can be compared to a list of known target genes for the specific transcription factor, if available. This allows the user to perform a quick comparison of his/her findings with what is already published or obtained from other sources, such as array analyses. Such comparisons provide bioinformatic validation of the ChIP experiment. A second comparison involves Gene Ontology (GO) IDs corresponding to the output list of genes. This list can be compared to a user's implemented list of GO IDs. This feature identifies genes associated with specific functions, processes, pathways or cellular components and allows extraction of specific genes from the TF Target Mapper list related to a specific function of interest. Gene and GO ID lists of interest can be uploaded using the parameters settings page.

### Use of TF Target Mapper – Example

**Figure 2:**
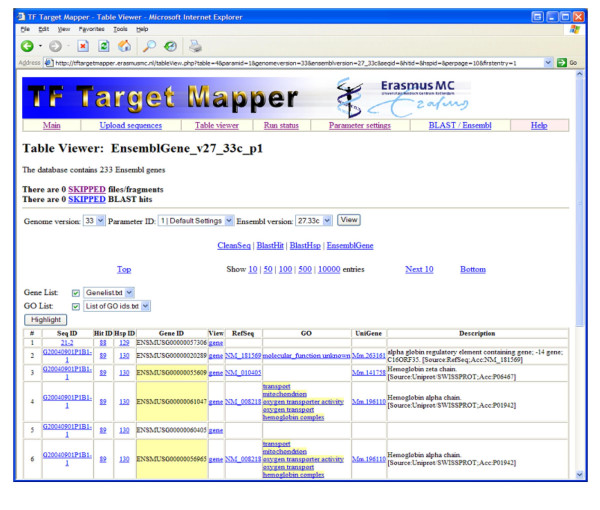
**Gene list output containing known targets of the transcription factor GATA-1** :  Example of a gene list output containing known targets of the transcription factor GATA-1 (*α *and *ζ globin*, highlighted in yellow). Information and links on the submitted sequences (seq ID), hit ID, HSP ID, Gene ID, contig/gene view, RefSeq, GO terms, Unigene information and description are depicted (HSP: High-scoring Segment Pair, fundamental unit of BLAST algorithm output representing local alignments with no gaps that achieve one of the top alignment scores in a given search, hit: general term containing one or more HSPs separated by gaps located in proximity in the sequence, GO: Gene Ontology).

We tested TF Target Mapper with randomly chosen sequenced clones from ChIP experiments using antibodies against the hematopoietic transcription factor GATA-1. This example demonstrates the utility and speed of this tool: The processing of 95 sequences and the extraction of annotated information on 372 genes 50 kb upstream and downstream of each hit took 27 minutes. Among these genes, known targets of GATA-1, e.g. *α-globin *and *ζ-globin *(Figure 2), were readily identified by comparing to a list of known GATA-1 targets, thus demonstrating the utility of this tool.

As a further test, we selected random sequences that contain hematopoietic transcription factor binding sites, as identified with TF Target Mapper (e.g. GATA-1, Sp1, CP2, NF-E2, LMO2). To assess if these sequences were real targets of GATA-1 (Additional File   2), we then performed ChIP (Additional File 3). Our preliminary data showed that most of these sequences were enriched in the GATA-1 immunoprecipitated material, thus increasing the possibility of them being real targets of GATA-1. These results further demonstrate the value of TF Target Mapper in identifying gene targets in chromatin immunoprecipitation approaches.

An increasing number of genomic ChIP approaches rely on the high throughput sequencing of sequence tags from cloned ChIPed DNA [15]. We therefore tested whether TF Target Mapper would be a useful tool for mapping short sequence tags. By default minimum sequence length required after the Clean-up procedure for a BLAST search to be initiated is 50 bp as specified by the 'Insert Threshold' parameter found on the 'Parameter Settings/Clean-up' page. This can be altered according to the needs of the user. When we tested sequences of 20 bp, TF Target Mapper was able to return hits. However the number of hits was high and this indicates the need for the implementation of a scoring system [15]. A system that could be adapted for this purpose has been developed recently (see [15] and also 'Discussion').

## Discussion

TF Target Mapper facilitates the bioinformatic analysis of libraries generated by cloning chromatin immunoprecipitated DNA. Whilst essentially developed for this purpose, TF Target Mapper is a tool of general utility that can be used with any set of sequences that require the extraction of specific information in a window around a BLAST hit against a known genome. A useful feature is that it allows the user to easily repeat the BLAST searches when a new genome version is released and to compare the results on the annotated information around each hit in between versions.

ChIP assays result in high background due to non-specific binding of DNA. Whereas recent experimental approaches have been developed aimed at reducing the background prior to cloning the ChIPed DNA (e.g. [15]), a useful feature that could be implemented in TF Target Mapper in future, would be the introduction of a scoring system that would take into account the frequency with which a specific sequence occurs in the ChIP library and the number of hits after BLASTing for a particular sequence in the genome [15].

TF Target Mapper was mainly used and tested with the mouse genome and we are presently expanding it for the human genome. It can also be expanded to include any of the other genomes in the Ensembl database. The utility of this tool will extend to the analysis of clusters of transcription factor binding sites in the wider area around each BLAST hit and implementation of other databases (e.g. microarray expression data), allowing for better prediction of real target genes.

## Conclusion

We devised TF Target Mapper, a BLAST search tool for the automatic extraction of annotated information on genes around chromatin immunoprecipitated sequences. We tested and demonstrated the efficiency of this tool with sequences bound in vivo by the hematopoietic transcription factor GATA-1. We anticipate that TF Target Mapper will contribute to the comprehensive bioinformatic transcriptome/regulome analysis aimed at investigating gene regulation. It can provide insights into the mechanisms of action of specific transcription factors and help elucidate the metabolic and developmental pathways these factors regulate.

## Availability and requirements

### Project name

TF Target Mapper.

### Project home page



### Operating system(s)

For use: Standard WWW browser (Mozilla/Firefox/I.E.); For server: GNU\Linux or Irix (tm SGI).

### Programming language

PHP, SQL, Perl, BioPerl.

### Other requirements

Ensembl & Bio Perl APIs, Perl, RepeatMasker, Cross_Match, MySQL database server, PHP-enabled Web server (e.g. Apache), NCBI Blast. Locally available NCBI formatted Mouse Genome sequence.

### Licence

ErasmusMC license is needed for people that wish to obtain the code.

### Any restrictions to use by non-academics

License needed.

## Abbreviations

TF: Transcription Factor.

ChIP: Chromatin Immunoprecipitation.

BLAST: Basic Local Alignment Search Tool.

GO ID: Gene Ontology Identity.

HSP: High-scoring segment pair.

## Authors' contributions

SH generated the code, the web interface and tested TF Target Mapper. MJM worked on the visualisation of hits on the chromosome ideograms and on the help pages, made contributions with ideas and was involved in critically correcting the manuscript. VCLdJ provided a template for the web interface, offered support concerning computer system maintenance and made contributions with ideas. PvdS has given support and guidance for the bioinformatic part of the project. FG and JS have made contributions with ideas to the project and were involved in revising and critically correcting the manuscript. EZK carried out the experiments for generating the sequences analysed, designed and supervised the project, tested TF Target Mapper and wrote the manuscript.  All authors read and approved the final manuscript.

## Supplementary Material

Additional File 1 TF Target Mapper application analytical flowchart : Analytical flowchart of the TF Target Mapper application including all its functions (RE: Restriction Endonuclease, TF: Transcription Factor). Click here for file

Additional File 3 Chromatin immunoprecipitation (ChIP) : Description of the ChIP method. Click here for file

Additional File 2 Chromatin immunoprecipitation (ChIP) to confirm sequences analysed as GATA-1 targets : Chromatin immunoprecipitation (ChIP) experiments with GATA-1 antibodies to confirm sequences analyzed by TF Target Mapper as GATA-1 targets. Semi-quantitative PCR was used with primers specific for sequences that were found by TF Target Mapper analysis to contain binding sites for hematopoietic transcription factors. The control experiments refer to ChIP performed with rat IgG, whereas GATA-1 ChIP assays were performed with the GATA-1 N6 rat monoclonal antibody. Input refers to DNA from formaldehyde crosslinked sonicated chromatin. It can be seen that most of the sequences tested (with the only exception of the sequence G) were enriched by the GATA-1 antibody compared to the control. The chromosomes where the sequences map are also depicted (chr: chromosome). Click here for file
