# Supplementary material for: TF Target Mapper: A BLAST search tool for the identification of Transcription Factor target genes
Source: BMC Bioinformatics. 2006 Mar 8;7:120. doi: 10.1186/1471-2105-7-120 (PMC1523221; doi:10.1186/1471-2105-7-120)
Supplement: Additional File 1 — TF Target Mapper application analytical flowchart : Analytical flowchart of the TF Target Mapper application including all its functions (RE: Restriction Endonuclease, TF: Transcription Factor). [file 1471-2105-7-120-S1.ppt]

## Slide 1
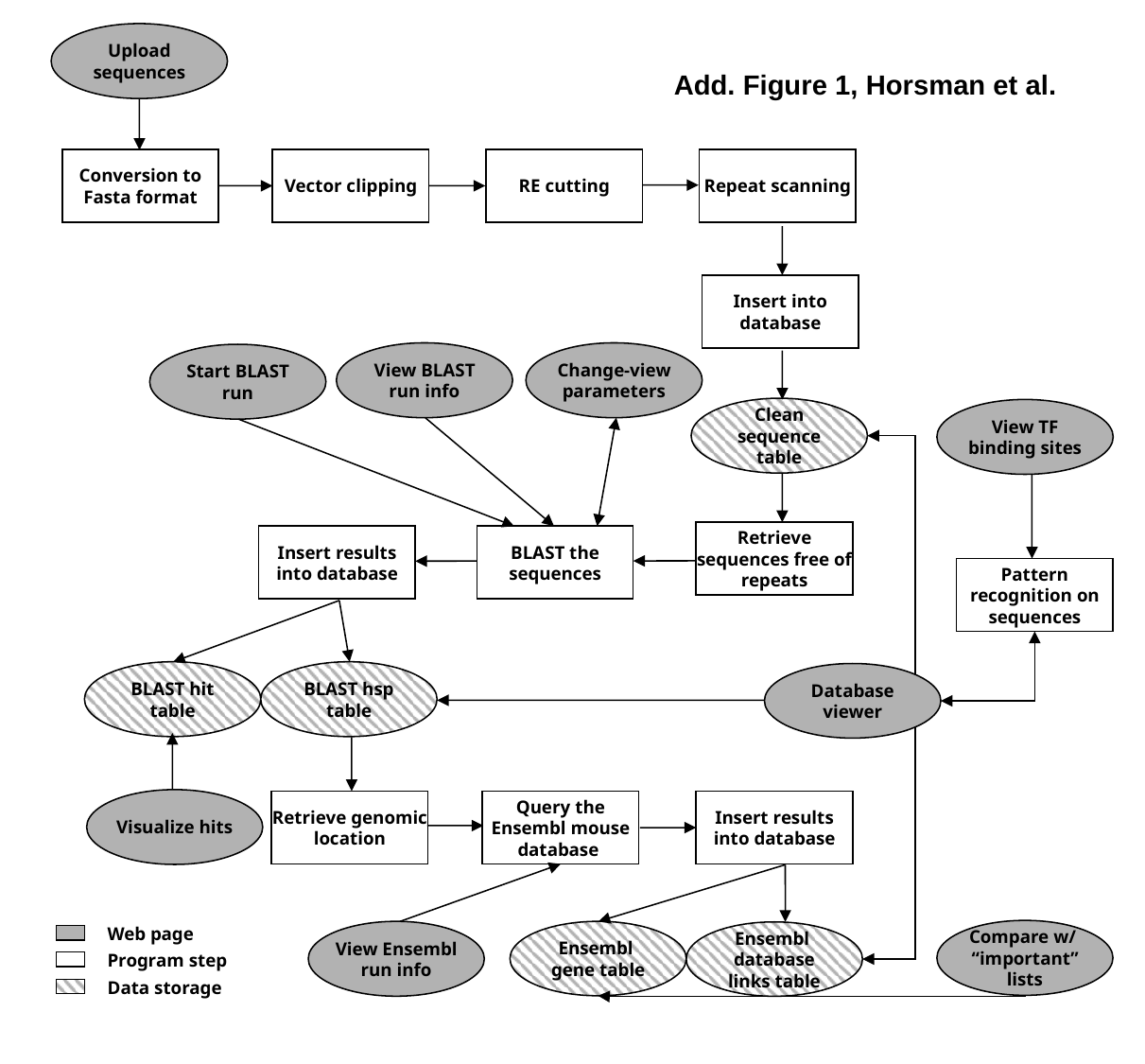

Upload sequences
Add. Figure 1, Horsman et al.
Conversion to Fasta format
Vector clipping
RE cutting
Repeat scanning
Insert into database
View BLAST run info
Change-view parameters
Start BLAST run
Clean sequence table
View TF binding sites
Retrieve sequences free of repeats
Insert results into database
BLAST the sequences
Pattern recognition on sequences
BLAST hit table
BLAST hsp table
Database viewer
Visualize hits
Retrieve genomic location
Query the Ensembl mouse database
Insert results into database
Compare w/ “important” lists
View Ensembl run info
Ensembl
gene table
Ensembl
database links table
Web page
Program step
Data storage
