# Supplementary material for: TF Target Mapper: A BLAST search tool for the identification of Transcription Factor target genes
Source: BMC Bioinformatics. 2006 Mar 8;7:120. doi: 10.1186/1471-2105-7-120 (PMC1523221; doi:10.1186/1471-2105-7-120)
Supplement: Additional File 3 — Chromatin immunoprecipitation (ChIP) : Description of the ChIP method. [file 1471-2105-7-120-S3.doc]

### Additional Methods – Chromatin immunoprecipitation (ChIP)

Preparation of crosslinked chromatin (2x10^7^ induced MEL cells treated with 0.4% formaldehyde for 10 minutes at room temperature), sonication to 300-800 base pair fragments and immunoprecipitations were as described in the Upstate protocol (www.upstate.com). Anti-GATA-1 protein-DNA immunocomplexes were immunoprecipitated in an additional step with an AffiniPure rabbit anti-rat antibody (Jackson ImmunoResearch Laboratories, West Grove, PA). GATA-1 N6 rat monoclonal antibody was obtained from Santa Cruz Biotechnology (cat. # sc-265).
