# Supplementary figures and images for: TF Target Mapper: A BLAST search tool for the identification of Transcription Factor target genes
Source: BMC Bioinformatics. 2006 Mar 8;7:120. doi: 10.1186/1471-2105-7-120 (PMC1523221; doi:10.1186/1471-2105-7-120)

## Slide 1
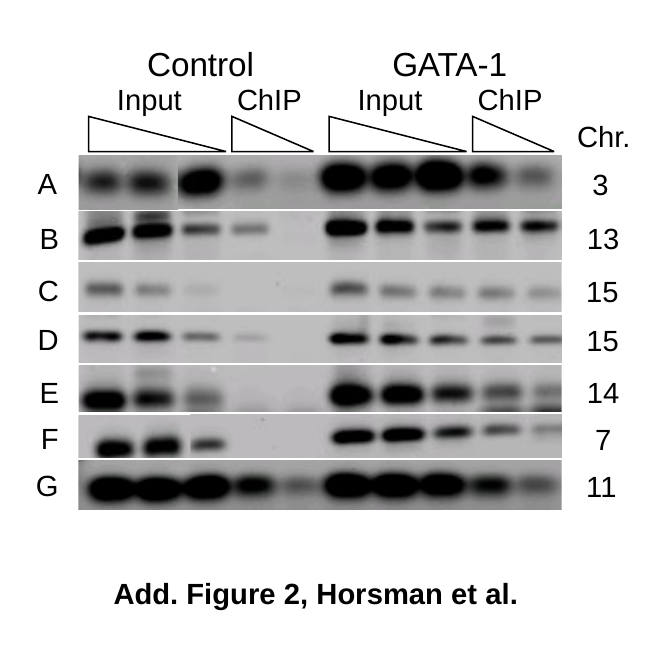

Control
GATA-1
Input
ChIP
Input
ChIP
Chr.
A
3
B
13
C
15
D
15
E
14
F
7
G
11
Add. Figure 2, Horsman et al.

Supplement: Additional File 2 — Chromatin immunoprecipitation (ChIP) to confirm sequences analysed as GATA-1 targets : Chromatin immunoprecipitation (ChIP) experiments with GATA-1 antibodies to confirm sequences analyzed by TF Target Mapper as GATA-1 targets. Semi-quantitative PCR was used with primers specific for sequences that were found by TF Target Mapper analysis to contain binding sites for hematopoietic transcription factors. The control experiments refer to ChIP performed with rat IgG, whereas GATA-1 ChIP assays were performed with the GATA-1 N6 rat monoclonal antibody. Input refers to DNA from formaldehyde crosslinked sonicated chromatin. It can be seen that most of the sequences tested (with the only exception of the sequence G) were enriched by the GATA-1 antibody compared to the control. The chromosomes where the sequences map are also depicted (chr: chromosome). [file 1471-2105-7-120-S2.ppt]
